# The COVID-19 Outbreak in North Africa: A Legal Analysis

**DOI:** 10.1177/0021909620983586

**Published:** 2021-11

**Authors:** Francesco Tamburini

**Affiliations:** Università di Pisa, Italy

**Keywords:** Covid-19, Maghreb, North Africa, state of emergency, transition to democracy

## Abstract

North African nations, especially Egypt, Algeria, and Morocco, have been heavily affected by COVID-19 if compared to other African countries. Governments in North Africa took proactive legal measures to manage the virus threat, safeguarding population health, but also triggering repressive and invasive mechanisms that in some cases jeopardized basic freedoms and rights. This work will analyze comparatively the anti-COVID-19 legislations, pointing out how the legislative measures mirrored the level of transition of democracy, the opacity of some regimes, exploitation of the pandemic to foster repressive control, and highlighting the weakness of new democratic institutions unprepared to balance health security and democracy.

## Introduction

Over the ages, the Arab world experienced many major epidemic diseases and pandemics. In classical Arabic, the term *ṭā′ūn* (طاعون) means plague, while *wabā’* (وباء) is the word for epidemic or pestilence. *Wabā’* can be described as an adverse change in the atmosphere, corruption to the substance of the air (Conrad, 1982; Dols, 1974), something that fits COVID-19 well. It might be considered the global event of this century. Some Islamic scholars have pointed out how the Quran foresaw the Coronavirus pandemic in the verses of the *sūra 74* Al-Muddaṯṯir (The One Enveloped) (Khenenou et al., 2020). The pandemic of the *fīrūs qūrūnā* (فيروس كورونا) quickly spread to all the continents in 2020. Arab countries have not been excepted. However, the Muslim Holy Book could not predict the profound impact of the virus on society, economies, and politics. North Africa recorded a relatively low incidence of the deadly pathogen among its population in comparison to countries on the northern shore of the Mediterranean.

Nevertheless, North African countries, especially Egypt, Algeria, and Morocco, have been heavily affected by COVID-19 (*Qūfīd-19*,19-كوفيد) if compared to other African nations. However, it is essential to recall that the official figures can be misleading because they depend on many factors, such as government transparency, strategy, and screening capacity. Hence, the number of cases could be underestimated.

**Table 1.** COVID-19 infections and deaths in North Africa (updated to 18 October 2020).

COVID-19 Infections and Deaths in North Africa (Updated until 18 October 2020)		
	Infections	Deaths
**Morocco**	173,632	2928
**Egypt**	105,424	6120
**Algeria**	54,402	1.856
**Libya**	48,790	725
**Tunisia**	40,542	626
**Mauritania**	7,608	163
**Western Sahara**	10	1

Source: www.who.int

**Figure 1.**
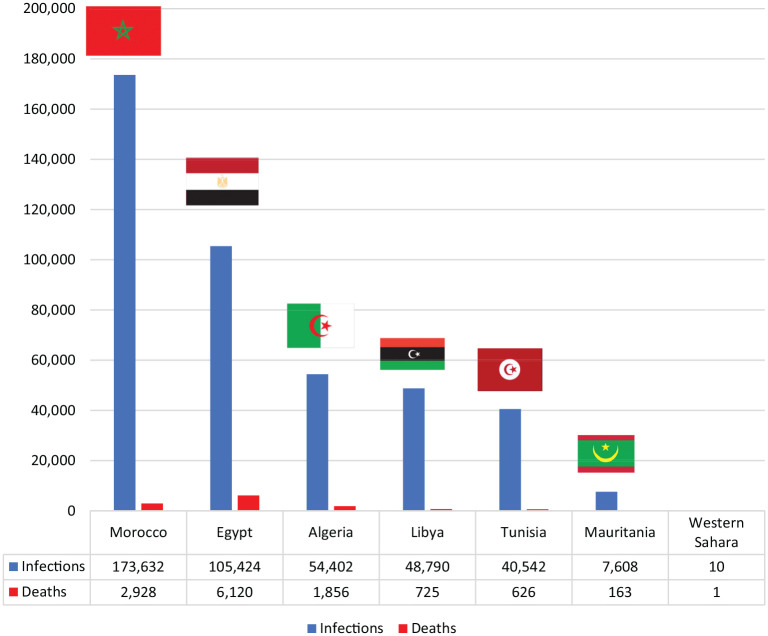
COVID-19 infections and deaths in North Africa (updated to 18 October 2020).

In any case, the epidemic caused medical prescriptions and behaviors which influenced the attitudes of the North African communities, as well as affecting their social and economic life. The North African region had already been experiencing pre-crisis challenges and deep-seated issues, such as weak economic growth, tricky consolidation of the democratic process, rapid population growth, widespread popular unrest, and in the case of Libya, even an ongoing bloody civil war.^1^ In this framework of unstable societies and political systems, rulers and presidents in North Africa acted very differently in handling the health emergency. However, all of them took extraordinary measures to manage the threat and safeguard the well-being and the health of the population. Nevertheless, these measures also highlighted the resilience of authoritarian rule as well as the different levels of transition to democracy.

Since the control of disease also means controlling the citizens, the additional powers granted to address the exceptional situation allowed some states to restrict certain human rights, silence the critics and in some instances sideline the judges, all beyond the requirements of legality, non-discrimination, proportionality, and necessity (Greene, 2018). Therefore, the emergency posed by COVID-19 has stressed political and institutional risks, revealing the danger of turning fragile democracies into everlasting hybrid regimes or liberalized autocracies. Authoritarian regimes could consolidate their grip on power. The United Nations is aware of this danger and warned that COVID-19 must not be an excuse for unlawful deprivation of liberty. That prohibition of arbitrary detention is absolute, even during times of public emergency (Office of the United Nations High Commissioner for Human Rights, 2020). The UN Secretary-general, António Guterres, raised the alarm about possible human rights violations due to government measures to fight COVID-19, warning that the health emergency was ‘a human crisis that is fast becoming a human rights crisis’ (Guterres, 2020).

This article will comparatively analyze the different legislation issued by the governments to cope with the emergency. Additionally, it will point out how the legislative instruments that were adopted in North Africa to control the pandemic can be used as a litmus test in the development of the transition of democracy. It may also act as a mirror of the impenetrability of some regimes that exploit the pandemic to expand state surveillance, toughen repression, foster democratic backsliding, and erode the mechanisms of accountability. The anti-COVID-19 measures could be the ‘tombstone’ to what remains of the fruits of the Arab Spring, but also the last chance for Arab governments in North Africa to show that something has changed since 2011.

## Egypt: The virus as a new ‘Trojan horse’ for authoritarianism

With a growing population of over 100 million people who mostly live in high-density areas, Egypt is the African state with the second largest number of fatalities after the Republic of South Africa, and the third largest in terms of the number of diagnosed COVID-19 cases, after the Kingdom of Morocco. Nevertheless, infection numbers may fall short in accuracy, and the total number could be up to ten times higher than official data suggest. The recorded data reflect the infections reported to the authorities. However, many patients with mild symptoms went untested and, therefore, unregistered, turning into ‘silent carriers’ of the disease. The same can be said of the reported figures on the loss of human lives because the al-Sisi government counts deaths that took place in hospitals. The demographic, with over 100 million inhabitants living on about 5% of the land, makes it extremely hard to practice social distancing and not to aggravate the spread of the virus.

The first case was reported on 14 February 2020, and the first death on 8 March (a German tourist in Hurghada, on the Red Sea coast). The first preventive measures included a lockdown and a curfew from 9 p.m. to 6 a.m. (ministerial decree 768, 24 March).^2^ Afterwards, the parliament passed a law that imposed fines of up to 4000 Egyptian pounds (250 USD) for not wearing a face mask in public spaces and also regulated the burial procedures for Coronavirus victims.

It was not difficult for the Egyptian government to control the situation in the country, maybe because it was already under firm control. Egypt has been living under a state of emergency for an extensive period, and the exercise of emergency powers has been far from exceptional. Most Egyptians have always lived under a constant state of emergency. The familiarity with such draconian measures aided the government in imposing the quarantine for COVID-19. The Republic of Egypt has cultivated a sort of reverence for special laws since its independence. From 1939 to 1945 the Egyptian government ruled under martial law, *al-ḥukāmi al-*ʾ*urfīyya* (الأحكام العرفية), which was imposed again in 1948, with the outbreak of the first Arab-Israeli war. The martial law lasted until 1950, but it was declared several times after that: from 1952 to June 1956, and from November 1956 to 1958 (Greene, 2018; Seif al-Islam, 2002). Martial law was abandoned in 1958 for a more comprehensive and strict type of legislation: Law concerning the State of Emergency (حالة الطوارى), 162/1958 *al-Qānūn bi šaʾn ḥalāt al-ṭawāri*. Law 162/1958 is very important because all the states of emergency declarations have been made in accordance with this law until today. The state of emergency was immediately enacted in 1958 and lasted up to 1964, and once again from 1967 to 1980. After Sadat’s assassination in 1981, law 162/1958 was almost continuously renewed (Reza, 2007). The Mubarak regime disregarded the rule of law, restricting fundamental freedoms and concentrating power in his own hands, again through the disputable law of emergency. It was repeatedly extended every three years until 2012, after the protests of the Arab Spring, when it was lifted after 31 years (Eldarak, 2012). Nevertheless, after the ousting of the ‘last pharaoh’, the situation did not change, and all hopes for a new democratic regime faded away.

The state of emergency was reimposed in April 2017, following the deadly bombings by Islamic terrorists that targeted two Coptic churches. The state of emergency has been enshrined in the constitution since 1971. The President of the Republic has the power to declare it, even if the *ḥalāt al-ṭawāri* is ‘for a limited period’, and the declaration is subject to ratification by the parliament – the People’s Assembly. The amendments to the 1971 constitution, which were enacted in 2014, retained this prerogative in article 154 (Serodio, 2017: 101).

Due to the extraordinary and controversial powers given to the president, the *ḥalāt al-ṭawāri* or state of emergency curbed any democratic movement in Egypt. The president can ‘by oral or written order’ restrict people’s freedom of assembly, movement, residence, or passage at specific times and places. He can arrest or detain suspects and allow searches of persons and places without any restrictions. He can also seize journals and publications; determine the opening and closing times of shops; confiscate any property; withdraw arms licenses; regulate the means of transport and evacuate regions, or cut off transportation between areas (*al-Qānūn*, 1958).

When the COVID-19 crisis broke out, law 162/1958 was still significant (it was extended on 20 January 2020 by Presidential decree number 20/2020), and nothing seemed to fit better to the situation created by the virus. Article 1 of law 162/1958 affirms that the State of Emergency may be declared whenever public security or public order is endangered, whether due to the occurrence of war, public disasters, disturbances, or the spread of pandemic *wabā’* (وباء). The *wabā’* was the ‘perfect storm’ for the al-Sisi government. After the wide spreading of the virus in the country, the regime could control the society and the legislative tools of the 162/1958. This law was amended on 6 May 2020, by law 22/2020, which gave new and more potent powers to the executive: the military has the power to investigate crimes; the competences of Courts were modified in order to include military judges;^3^ the president has the right to ban private and public gatherings, close schools, universities, ministries, companies, and to compel Egyptian citizens who live abroad and are returning home to undergo quarantine measures; the government can impose restrictions on the exportation of certain goods and to control scientific research. Of the 18 amendments to the law, only 5 concern the public health emergency. The remaining 13 revisions could thus be enforced whenever declared and without any relation to the pandemic.

Moreover, many of the revised articles are in direct contradiction to the 2014 constitution. The President has the right to exercise the functions of the judiciary, while art. 184 of the constitution states that judicial power is independent, *al-Sultat al-Qaḍāiyyat mustaqilat* (السلطة القضائية مستقلة) (Zartner, 2014). The law now denies all the guarantees established in the Criminal Procedure Code during investigations, preventing the defendant from litigating under art. 97 (‘litigation is a safeguard right guaranteed to all’ - التقاضى حق مصون ومكفول للكافة), and art. 96 of the constitution (‘the accused is innocent until proven guilty in a fair court, which provides guarantees for him to defend himself’); Art. 54 states that detention has to be limited in time.

On the other hand, the new law bypasses this article, because it grants exceptional powers to a public prosecutor. He is not obliged to complete an investigation in a determined time, resulting in prolonged pretrial detentions; Art. 155 grants to the president only the possibility of exercising the right of pardon or mitigation of a sentence (رئيس الجمهورية بعد اخذ رأى مجلس الوزراء العفو عن العقوب), following the advice of the cabinet, whereas articles 13 and 14 of the law allow the head of state to amend, decrease, abolish or suspend the execution of sentences.

It must be emphasized that the amendments also extend the definition of ‘terrorist entities’, including media platforms, syndicates, and trade unions. It goes even further than the 2015 anti-terrorism law n. 94, which defined terrorism in such vague terms that it jeopardizes political and civil freedoms, as well as human rights, and the Law Organizing the Lists of Terrorists and Terrorist Entities (commonly known as the Terrorist Entities Law n. 8 of 2015).

As a result, enforced disappearance and the arbitrary detention of individuals increased under the newest version of law 162/1958. Between February and July 2020, Egyptian authorities have arrested at least 10 doctors and 6 journalists. Health workers have been warned not to discuss the health crisis with the foreign press or the media in general. The government led a campaign of harassment against medics and paramedics. They have been subject to threats and punitive measures just for daring to express their safety concerns or criticizing the regime’s handling of COVID-19 (Amnesty International, 2020). Those who talk about the state’s fragile health system or the shortage of protective gear are perfect targets of the government’s repression.

The use of the state of emergency goes beyond the virus crisis itself, and has been extended instead to stabilize the political leadership, which does not want to rely on popular legitimacy. The consequence was a further crackdown on opposition groups, the circumvention of human rights obligations and going beyond the rule of law in the name of public health. It can also be argued that the Egyptian government has been more concerned about its decreasing level of legitimacy and silencing those who criticize government responses to Coronavirus, than engaging in the fight against the virus itself.

## Algeria: A soft strategy in an inflexible system

The Algerian authorities confirmed the first COVID-19 case (an Italian businessman) on 25 February 2020. The toll rose to 139 on 21 March. The same day the government, led by President Abdelmadjid Tebboune, decided to issue the first legislative provision aimed at fighting the spread of the pandemic: executive decree 20-69. This law set up a framework of social distancing, *al-tabā′ed al-′aijtimā′eīa* (التباعد الاجتماعي), such as the closure of all drinking establishments, restaurants, and recreational areas, and the suspension of all public transport in the national territory. The governors (*awliyā*) of the administrative divisions were responsible for the enforcement of the executive decree and had the power to seize hotels and other public or private infrastructure, means of transport and personnel belonging to civil defence, health service or national security institutions (*Journal Officiel de la République Démocratique et Populaire* (JORADP), 2020a).^4^

In comparison to other similar laws in Europe or the Middle East and North African (MENA) region,^5^ decree 20-69 has taken a very light touch approach against the virus, which could be seen as ineffective (flights from severely affected countries such as France continued to arrive in Algeria). The minimalist approach of 20-69 also appeared to be respectful of civil liberties and the rule of law. First, 20-69 is an executive decree, which is promulgated by the prime minister, Abdel Aziz Djerad, according to article 99 of the constitution. This means that the government decided not to entrust such sensitive matters to a presidential decree that would have underscored the already extensive powers of the president of the republic. We should also consider the powers of enforcement given to local governors rather than the central Algerian state, which involved a degree of decentralization in the fight against the virus. Finally, it should be noted that the law and its special measures pplied for a time-limited period of only 14 days (article 2). The apparently ‘over-timid’ approach to the first anti-epidemic actions must be framed in the Algerian internecine political situation. After the perennial presidential mandates of Bouteflika, who ruled Algeria from 1999 to 2019, and the riots that took place in 2011 which put an end to the application of the 19-year-old state of emergency in February of the same year, the Algerian elite could not afford to use the ‘iron fist’. The proclamation by the president of the republic of the state of emergency or state of exception, according to art. 105 of the constitution, would have triggered protests and uprisings among the population. The constitution allows the president in cases of ‘urgent necessity’ – in Arabic *ḥalāt al-ḍarūra al-muliḥat* (حالة الضرورة الملحة) – to decree for a limited time a state of emergency –*ḥalāt al-ṭawāri* (حالة الطّوارئ) – or a state of siege – *al-ḥaṣār* (الحصار), and take all the ‘necessary actions’ – *al’ iiǧrā′āt al-llaḏimat* (الإجراءات اللازمة) – to restore the situation. Terms such as urgent necessity and necessary actions are so broad and blurry in their interpretation that they could in theory have allowed the Tabboune to use the pandemic as an alibi and set in motion article 105.^6^

The approach did not change even after the laws that followed 20-69. The Algerian government issued another executive decree on 24 March, 20-70, establishing ’complementary measures’ – *tadābīr takmīlīyya* (تدابيرتكميلية) – of prevention and fight against the spreading of the virus. Decree 20-70 provided a ‘system of domestic quarantine’ – *niẓām al-ḥaǧar al-manzilīyya* (نظام الحجر المنزلي) – for some regions (wilāyāt) that could be total, kull*īā* (كليا), or partial, *ǧuziīā* (جزئيا), depending on the epidemiological situation (artic;e 2) (JORADP, 2020b). The partial domestic quarantine required citizens not to leave their homes during stated hours; on the other hand, the total quarantine was a complete lockdown for private individuals. Out of 58 wilāyāt, only 2 were covered by the measures: The Berber *wilāya* of Blida had a total quarantine for 10 days (article 9), while the *wilāya* of Algiers, with over 3 million inhabitants, only had a partial quarantine, from 7 p.m. to 7 a.m. (article 10). There were administrative and penal sanctions for breaching the quarantine measures and social distancing (article 17). However, it is worth noting that, according to the law, the Algerian army had no part in upholding the measures. This is not a minor detail if we consider the traditional power held by the National Popular Army in the country. Police officers saw that the established rules were respected and that any gathering of more than two people was prohibited.

It would be easy for the government to declare a complete lockdown of the country, as many European countries did. Algiers preferred to apply a ‘variable geometry’ method, which followed the pandemic’s development. The partial domestic quarantine was extended to the other nine wilāyāt on 28 March under law no. 20-72 (JORADP, 2020c). When the number of positive cases and deaths grew in April, the government issued a new decree (no. 20-102) that broadened the *al-ḥaǧar al-manzilīyya ǧuziī*ā to all national territory for 15 days.

There was some distinction between the regions since the total quarantine of the *wilāya* of Blida was amended to a partial lockdown. Nine wilāyāt had different lockdown hours (from 5 p.m. to 7 a.m.) (JORADP, 2020d). The lockdown structure in the wilāyāt was prolonged and underwent minor changes in the ensuing months.^7^ However, the government did not modify the general approach. It did not want to ‘freeze’ the country with draconian measures, and much of the power was handed over to the governors, who could turn a partial lockdown into a total quarantine at their discretion.^8^ The Tabboune government did not want to paralyze Algeria, which was already suffering from structural, economic, and political problems that the virus had reinforced and multiplied. The political system, guaranteed by a government coalition between the Front de Libération Nationale and the Rassemblement National Démocratique, had lost its legitimacy years ago. The election of the new president Abdelmadjid Tebboune on 12 December 2019 did not bring any real change, and the younger generation no longer recognized the ruling political class.

It is undeniable that the pandemic itself helped the regime because it ‘emptied the streets’ and put a stop to the massive anti-regime demonstrations organized by the so-called *Ḥirak* (movement in Arabic) protests from February 2019 seeking the departure of all the system political leadership. In May 2020, many supporters of the ḥirak defied the ban on public gatherings and organized of massive anti-regime rallies in various towns, and anti-regime campaigns were held on the web, social media and even the radio (i.e. Radio Corona Internationale, founded on 23 March 2020). However, despite the outbreak of coronavirus (*fīrūs qūrūnā*), the regime did not stop its crackdown against the opposition and the media, using the usual tools to control society. The same tools and legislation helped the regime to survive. The only difference is that the grip on power was not implemented by ad hoc legislation due to COVID-19. A new state of emergency with superpowers granted to the president of the republic could have sparked widespread and more massive political protest than the *ḥirak* movement. The regime tried to reassert stability showing its ability to cope with the virus without overtly infringing civil liberties. On the other hand, many Algerians thought that the state’s response to the pandemic was a test of its efficacy on economic, political, and health issues. The mortality rate, the highest in the Maghreb area, showed the unpreparedness of the government, revealing that the 2,500 intensive care beds were just propaganda.

The low key approach to the crisis and the ‘variable geometry’ measures could have another interpretation. It might not be a conscious political move, but rather the incapacity of Algerian authorities to act when needed. The president was hesitant to take drastic measures and to discharge his responsibilities to the prime minister and the governors of the *wilāyāt*. Tebboune represents only a narrow majority of the Algerian electorate, due to the massive abstentions in the 2019 elections (60.12 %). Therefore, he lacks popular legitimacy not only to solve the crisis but above all to make difficult and unpopular decisions.

## The Moroccan ‘legislative recipe’ for the pandemic

The first confirmed cases in the Kingdom of Morocco were recorded on 2 and 3 March 2020 (two Moroccan citizens living in Northern Italy who had returned to their homeland a few days earlier). The first action taken by the coalition government^9^ led by Saad Eddine Othmani was to suspend all flights and ferryboats to and from Algeria, France and Spain, and to close all schools on 13 March. The pandemic quickly spread all over the country, turning Morocco in one of the most affected nations in the Maghreb. Morocco lacked specific legislation to cope with the situation; nevertheless, it was necessary to act quickly.

The Ministry of the Interior decided to declare, through an administrative procedure, a state of health emergency, or *ḥalāt al-ṭawāri al-ṣaḥīat* (حالة الطوارئ الصحية), on 19 March. Restriction of movement was introduced from the next day until further notice. The statement aimed at the ‘preservation of health and security of the Moroccan society’ also detailed the exceptional quarantine measures introduced to keep the coronavirus under control, such as the closure of shops and the domestic isolation of all citizens. The prohibition of public and private means of transportation was declared two days later. All these procedures gave rise to severe concerns since many contended that a legal loophole existed, and a simple administrative act was not enough to guarantee fundamental freedoms and rights. According to the non-governmental organization the Centre d’Études en Droits Humains et Démocratie, established in Morocco in 2015, two laws were available: the 1967 royal decree 554-65 and article 8 of the 2001 *loi-cadre* (framework law) 34-09 (Centre pour la Gouvernance, 2020). Royal decree 554-65 required notification of the presence of contagious or epidemic diseases by any member of the medical profession to the public authorities. The medic should be prescribing prophylactic measures to control infections (*Bullettin Officiel du Royaume du Maroc* (BORM), 1967).

Decree 554-65 was, however, unfit for the COVID-19 pandemic (for example, the decree could only sanction the non-application of the rules to members of the medical staff and not to citizens). Article 8 of the law 34-09, on the other hand, stated that in the case of transmissible diseases and dangers to the community, public health services had to submit the sick person, and the persons in contact with him/her, to appropriate care and prophylactic measures, according to existing legislative provisions (BORM, 2011). Although this law could have been associated to the International Health Regulations, the law was judged incomplete and untrustworthy in the complicated situation of the pandemic; the law was issued in 200518 and signed by the Kingdom of Morocco in 2009. Of course, the lack of a real legislative basis to find any legal measure was well known by the government, who had, on the other hand, the challenging task to take immediate action to limit the mounting wave of the virus. The real problem with the Moroccan legal system was that it did not include the notion of a ‘state of emergency’: it only included a ‘state of exception’, *ḥalāt al-āstaṯniā* (الحالة الأستثناء). The state of exception had been used several times by King Hassan II to suspend the constitution, dissolve the parliament, concentrate executive and legislative powers in his own hands, and to allow him to head the government without a prime minister (Berrahou, 2002; Vermeren, 2010). The state of exception was enshrined for the first time in the 1963 Moroccan Constitution, and was repeated in all the following constitutions until the last Constitutional Chart of 2011. When Mohammed VI succeeded his father Hassan II, he started liberalizing and upholding reformist projects and introduced unprecedented checks and balances to the political system, while giving more robust powers to the legislative branch to oversee government policy (Melloni, 2013: 5-17; Vermeren, 2009). The state of exception did not appear to be the best way to face the challenge of the virus, practically and politically. The king could use a royal decree to issue the ḥalāt al-āstaṯniā, or *ẓahīr* (ظهير), which is a King’s discretionary act that cannot be called into question by any institutional authority and which have no place in the Moroccan hierarchy of law sources. Nevertheless, Mohammed VI preferred to use less discretionary forms of legislation.

Despite the emergency, the declaration of a state of exception would have represented a retrograde step for the Moroccan population. Besides, the *ḥalāt al-āstaṯniā* itself did not fit well with the pandemic crisis. According to article 59 of the 2011 constitution, the state of exception can be declared ‘when the integrity of the National territory is threatened or in case of events obstruct the regular functioning of the constitutional institutions’ (Melloni, 2013). Therefore, article 59 could only be applied by creative interpretation of the constitution. The state of siege, *ḥalāt al-ḥaṣār*, declared by the King with a *ẓahīr*, countersigned by the head of the government (article 74), was also deemed inappropriate under the current circumstances. The constitution did not specify the exact meaning of a state of siege. Comparative experiences suggest that this is an exceptional and temporary state declared by the government in the face of imminent national danger, with the intention of preserving public order. Even in this case, the government decided not to use the juridical tool offered by the constitution. It could be the reason why the Othmani government, endorsed by the King, decided to follow a different juridical path and created an entirely new legal system.^10^ It was created through a decree-law, *marsūm biqānūn* (مرسوم بقانون), a sort of framework law based on the health emergency. General obligations and principles were laid down. The governing authorities, however, were tasked with enacting further legislation with specific measures. It was coined decree-law 2-20-292 ‘enacting special provisions to the State of Health Emergency’, issued on 23 March 2020 (BORM, 2020b: 506). In essence, it was an unprecedented measure for the Moroccan legislation, which developed the concept of a state of health emergency for the first time.^11^ According to article 1, a state of health emergency can be proclaimed partly, or throughout the national territory, every time the lives and security of people are threatened by the spreading of contagious or epidemic diseases, and when it is necessary to take urgent measures to prevent their diffusion. This new form of a state of emergency can be proclaimed only after a joint proposition by the ministers of the interior and health (article 2). The law prescribes that all citizens living within the areas where the health emergency is proclaimed must comply with the requirements adopted by the public authorities. Breaching of these regulations can lead to both imprisonment (from one to three months) and fines (from 300 to 1500 dirhams).

Decree 2-20-292 was presented as a general decree-law, but it had been explicitly studied for COVID-19. It is no coincidence that the decree quotes “contagious or epidemic diseases”, *’amrāḍ mu’edīat wa wabā’* (أمراض معدية أو وبائية). The aim was, however, to maintain the compromise between the preservation of public freedom and the maintenance of public order and safety. In other words, it provides the use of exceptional or outstanding legislative measures, but always within the framework of legality. For this reason, the technical procedure used to enact the state of health emergency and the safeguards behind it must be noted.

Firstly, the Moroccan legislator decided to adopt the decree-law instrument, and not the *ẓahīr*, which belongs exclusively to the King, as it is an uncontrolled legislative act. The preamble to 2-20-292 reiterates how the decree has been issued abiding by articles 81, 21 and 24, paragraph 4, of the constitution. Article 81 enabled the government to issue decrees, which must be approved by the parliament.^12^ This approach can be interpreted as one of the marks of the decisive evolution and consolidation of democratic transition in the North African kingdom. In other words, in taking such significant legislative actions the government was responsible for a transfer of powers from the King to the government under the 2011 constitution which changed traditional perceptions of the reigning and ruling King. Mohammed VI still holds firmly to his political powers. However, he also showed that in a time of crisis, he could afford to entrust important decisions to the elected government, while respecting the formal procedures of the constitution and parliament.

Article 21 states that ‘All have the right to the security of their person and of their kin and to the protection of their assets. The public powers assure the security of the population and of the national territory within respect for the fundamental freedoms and rights guaranteed to all’. This reference is essential since it establishes the principle whereby in the state of health emergency ‘fundamental freedoms and rights’, *al-ḥurrīyyat wa al-uqūq al-asasīyyat*, (الحريات و الحقوق الاساسية), are guaranteed. It means that regardless of the gravity of the health situation, the measures taken should not endanger the fundamental rights of Moroccan citizens. This is of utmost relevance in a country where during the so-called ‘years of lead’ under the rule of Hassan II, fundamental rights and freedoms were flagrantly violated in the shadow of the ‘state of exception’.

In paragraph 4 of article 24 it is stated that ‘The freedom to circulate and to establish oneself on the national territory, to leave it and to return, is guaranteed to all, in accordance with the law’. This article has been placed in the decree-law to point out that limitation of the freedom of movement, which is endorsed by the constitution, can be limited by the law (2-20-292 itself). It may seem a redundant concept, but the legislator aimed to point out that all the measures tending to restrict the movement of citizens are in any case foreseen by the constitution when it states ‘in accordance with the law’, *wafq al-qānūn* (وفق للقانون).

Decree 2-20-292 set up a framework for a new law that could specifically cope with COVID-19, and the government issued decree 2-20-293 on 24 March 2020, just one day after the abovementioned decree-law. The decree entitled ‘Declaration of the State of Health Emergency on the whole national territory to face the spreading of the Coronavirus - COVID-19’ was signed by the prime minister and countersigned by the minister of the interior, Abdel Ouafi, and the minister of health, Khalid Ait Talib (BORM, 2020b: 507-507). The Moroccan government decided to act decisively and impose a state of health emergency throughout the national territory, *arǧā al-turāb al-waṭanīyya* ( ارجاء التراب الوطني), but for a limited period (until 20 April) (Article 1). The public authorities (*ʾawliyā*^13^ in the regions, and governors in the provinces and prefectures) had the duty to take all the necessary measures to ensure that the quarantine was respected and that citizens did not leave their homes. The government, however, did not hesitate to parade the armoured vehicles of the *Force Armée Royale* in the streets. The only exceptions to domestic isolation were in cases of extreme necessity: travel from home to the workplace for essential public services and liberal professions in the critical sectors; to buy essential goods; for urgent family reasons; and to go to medical practices or hospitals. Moreover, all public gatherings and meetings were forbidden.

Except for the ‘false start’ represented by the administrative procedure of 19 March, which gave rise to many constitutional doubts, the legal instruments adopted by the Moroccan government in this major health crisis showed uncompromising attention to the formal proceedings of democracy enshrined in the 2011 constitution. Even though the kingdom is far from being a modern parliamentary monarchy, it found a happy medium between internal security, health issues, and respect for the rule of law and human rights. Of course, not everything was perfect in the first month of the pandemic. Between March and April, 25,857 persons were prosecuted for violations of the quarantine, including many opponents of the government. Networks and social media relayed allegations of ill-treatment committed by law enforcement officers responsible for ensuring compliance with confinement and a crackdown on any form of dissent. Over 91,000 Moroccans have been prosecuted for breaching the lockdown, and among those arrested were human rights activists and journalists (Moceri, 2020). The use of force could be reiterated in the future, once the health emergency is over, and contribute to the normalization of strict security practices as well as the institutionalization of state control on civil society movements in public spaces. This scenario is not impossible given the pre-existing social tensions and government repression of activists existing before the pandemic.

However, if we focus only on the rule of law, we should point out that the main flaw in the legal procedure was the delay in ratification by the parliament of decree-law 2.20.292, as required by article 81 of the constitution. The state of health emergency was extended on 18 April for one more month. Nevertheless, the House of Representatives (*Maǧlis al-Nuwāb*) and the House of Councilors (*Maǧlis al-Mustāsharīyyan*) approved the extension on 24 April, and 6 May respectively.^14^ It means that the adopted measure had been lacking a legal basis for about a month. Therefore, the ‘legal management’ of the health emergency was not up to the ‘legal engineering’ (Boukhima, 2020).

## The Tunisian ‘disappointment’

Elyes Fakhfakh became prime minister in Tunisia on 27 February 2020. He was the eighth head of government since the 2011 revolution that ousted President Zine El Abidine Ben Ali and transformed Tunisia, one of the most successful outcomes of the Arab Spring in terms of the transition to democracy (Masri, 2017). Fakhfakh and his precarious coalition government found themselves coping not only with political instability, Islamist radicalism, regional disparities, corruption, and a growing economic crisis, but also with the unexpected challenges of COVID-19. The health crisis represented one of the biggest hurdles for the newly born Tunisian democracy, which might be swept away by the pandemic. The exodus of skilled medics and para-medics in 2019, and the extremely outdated health infrastructures (just 331 intensive care unit beds nationwide, 49 of which were out of use) were likely to plunge Tunisia into an abyss.

The first reported case of infection on 2 March 2020 was a Tunisian citizen who had just returned from Milan. The growing number of COVID patients that followed forced Tunisian institutions to face this unprecedented emergency. Paradoxically, Tunisia has been in a state of emergency almost since January 2011, when the decree 2011-184 proclaimed a state of emergency on the entire Tunisian territory (JORT, 2011). This decree, which made specific reference to law 78-50, was the first of a long series of similar legislative acts that prolonged or proclaimed the states of emergency up to the present day. In view of a series of complex events, such as the social unrest due to the difficult transition, political assassinations, terrorist attacks of Sousse and Bardo in 2015 and the general instability of the region, all the Tunisian governments decided to extend the *ḥalāt al-ṭawāri* indefinitely. The last presidential decree (2020-54), issued by President Kaïs Saïed on 24 May 2020, prorogued the state of emergency until November 2020. Hence, the long-running state of emergency became normality. The problem with these decrees is that they all make a precise reference to law 78/50 regulating the state of emergency. This law was developed in January 1978 during the Bourguiba regime (1956–1987). Article 1 stated that the state of emergency could be declared on all, or part, of the territory of the republic, either in the event of an imminent danger resulting from severe attacks to public order or in the case of events perceived as public calamities in view of their gravity. The state of emergency enabled the governors of the provinces to forbid the movement of people or vehicles. Any strike or lockout decided before the declaration of the state of emergency regulated the residence of citizens. The minister of the interior had the power to issue restricted residence for citizens whose activity was dangerous to public safety and order, and to demand the temporary closure of theatres and meeting places of all kinds. He could order searches and take the necessary precautions day and night to ensure the control of the press and publications of all sorts (JORT, 1978).

The state of emergency was deemed unsuitable against the coronavirus. Thus it was neither ‘upgraded’ to the new situation, nor lifted. Apart from the 1992 law, 92-71, on ‘transmissible diseases’, there was nothing in the Tunisian legal arsenal. The Fakhfakh government used this law to enhance the Tunisian legislation. Governmental decree 2020/152 of 13 March defined COVID-19 as a transmissible disease making coronavirus comparable to others pathologies on the list of diseases set out in the original decree 92/71. Therefore, the government could declare an epidemiological crisis and even envisage possible sanctions in case of breach of future prophylactic measures (article 8). It is not an exaggeration to say that all the legislative measures issued by the Tunisian authorities derive from decree 2020/152.

President Kaïs Saïed issued two presidential decrees that imposed a curfew, *bimane al-ǧūlān* (بمنع الجولان), on all national territory from 18:00 to 6:00 (JORT, 2020a). He expanded the restrictions on movement and gatherings beyond the curfew times and banned meetings of more than three people in public spaces (JORT, 2020b). Decree 2020-28 established a complete lockdown on the country.^15^ The vital detail is that 2020-24 and 2020-28 were very harsh decrees (one containing four articles and the other just two). They did not contain detailed information about potential sanctions,^16^ which caused confusion among citizens and security forces and led to several arrests. The same lack of clarity affected presidential decree 2020-156 (22 March 2020) (JORT, 2020c) which listed exemptions to the limitation of movement. However, they were so imprecise that they led to confusion in their application and several cases where they were applied arbitrarily by the authorities (Ferchici et al., 2020).

However, decrees 2020-24 and 2020-28 did not mention any safeguards relating to the protection of fundamental freedoms and rights. They did not quote, for example, article 49 of the constitution, which contains limits on the restrictions of rights. On the other hand, both texts referred to article 80 of the 2014 constitution, which states that ‘in the event of imminent danger, *ḵaṭar wašīk* (خطر وشيك), threatening the nation’s institutions or the security or independence of the country, and hampering the normal functioning of the public institutions, the president of the republic may take any measures necessitated by the exceptional circumstances’. In accordance with the same article, the decision was taken by the president after consultation with the prime minister and the speaker of parliament, and the president of the constitutional court was informed subsequently of the decision. Moreover, 30 days after the entry into force of these measures, the speaker of the Assembly of the Representatives of the People or 30 of its members are entitled to apply to the constitutional court to verify whether or not the circumstances remain exceptional. Unfortunately, 6 years after its creation in the 2014 constitution, this independent judicial body remains vacant because its 12 members have not yet been elected.^17^ The result is that presidential decrees partly lack a legal basis, and there is no possibility of judicial recourse against exceptional measures taken by the authorities in case of conflicts of interpretation. The recourse to article 80, which is based on article 16 of the 1958 French constitution, meant, without openly stating it, the declaration of a state of exception, giving the president of the republic sweeping legislative and executive powers. In short, Tunisia found itself at the same time in a state of emergency and in a state of exception. The state of exception related to the COVID-19 emergency could also be called into question. Despite the danger of the pandemic and the importance of the precautionary measures, the health crisis did not hamper the functioning of the public institutions, as stipulated in article 80. The proof is that before and after the declaration of the curfew, the presidency of the republic, the government, the parliament, and other institutions continued to operate.

The virus outbreak highlighted the coordination between the two heads of the executive, the president of the republic and the prime minister, in controlling the health emergency. The head of state received sweeping powers according to article 80. On the other hand, the head of the government used article 70, paragraph 2, requesting authorization from the parliament to issue decree-laws of a legislative character for a limited period not exceeding two months, and for a specific purpose. The Assembly of the Representatives of the People granted permission on 4 April giving way to the law no. 2020-19 on 12 April. The permission of the Assembly of the Representatives of the People could set in motion a possible institutional short circuit with both the president of the republic and prime minister simultaneously asking for exceptional powers, quoting two different articles of the Constitution, articles 80 and 70.

In any case, law 2020-19 allowed Fakhfakh to issue decree-laws in four main fields: fiscal, financial and social; rights, freedoms and determination of crimes, such as acts that might spread the virus; health, education and environment; the functioning of public services and the private sector (JORT, 2020d). This wide range of legislative issues granted to the head of government, allows him to act quickly. However, it is not free from defects since it led naturally to a confusion of roles between the legislative and the executive branches. Moreover, there is no control by the constitutional court, and the parliament can supervise and approve the decrees only after two months. It could contribute to the imposition of a presidential system, where the legislative branch is subsidiary to the executive.

Moreover, law 2020-19 could also create confusion between the executive and judicial branches. In fact, in the framework of the law, as mentioned above, Fakhfakh issued the decree-law 2020-12 on 27 April adding a new article (141 bis) to the penal code, permitting, in case of imminent danger or to prevent transmissible diseases, the use of audiovisual communications in court hearings (JORT, 2020e). This law is intended to be active not only to cope with the COVID-19 emergency but also in other unclear circumstances. The official journal in French mentions ‘*danger imminent*’, but the Arabic version is even more ambiguous, referring only to a ‘state of danger’, *ḥalāt al-ḵaṭar* (حالة الخطر). It is not clear if it is a general danger weighing on the nation or a specific danger related to the defendant’s transfer from the prison to the courtroom.

Furthermore, is a flu epidemic sufficient reason to resort to audiovisual communications in hearings? Such sensitive issues should have received more attention in parliament. This is not a temporary measure, but an amendment to the penal code with severe implications for the proper functioning of justice and the protection of fundamental rights.

At the beginning of the pandemic, Tunisia faced a complicated double task: to cope with a health emergency and, at the same time preserve the rule of law and democratic transition after the revolution. The challenge has not been entirely met.

## Conclusion

It is not always recognized that COVID-19 is not only a significant health crisis but also a crisis for the legitimacy and survival of democracy and the rule of law in some parts of the world. In North Africa, democratic institutions received more attention after the ‘revolution’ of the Arab Spring in 2011. Some countries began a complex reformist agenda to implement democracy; others stayed in the ‘grey zone’ of hybrid regimes. Coronavirus has suddenly triggered a large number of exceptional legal responses from governments, forcing some of them to rethink, adapt or stop democratic reforms. The comparative examination of anti-COVID19 legislation in North Africa focused on these responses, taken in a desperate effort to contain the spreading of the virus as quickly as possible. The difference between legislations is in the way in which they resort to emergency powers or a state of emergency and how these are linked to the protection of fundamental freedoms enshrined in constitutional guarantees. For all the states, the real complexity was to create new laws to a tight and for a temporary emergency, while still respecting the constitutional framework. In the end, it was a ‘democracy stress test’.

Political spaces inevitably shrank because the lockdowns and social distancing measures made political rallies impossible. Moreover, the first months of the emergency (March and April 2020) witnessed a re-enforcement of the executive in all the governments and a substantial limitation of freedom. This also happened in well-established Western democracies, since controlling the disease also meant controlling the freedom of citizens. However, the situation in North Africa is much more complicated.

A cursory examination could only reveal that in autocratic regimes such as Egypt, or to a lesser degree Algeria, the pandemic exacerbated pre-existing problems. It provided a different alibi for limiting civil liberties, curtailing media freedom, cracking down on dissenters, and dismantling the last bastions of checks and balances. On the other hand, countries that had made strides in democratization over the past years like Morocco and Tunisia fared better and were less likely to have governments who abused pandemic-related hyper powered executives. Nevertheless, only the Egyptian situation matches with this simple scheme. Egyptian response to the crisis followed the same path of abuse of extra-constitutional powers and systemic repression of opposition and freedom of speech, which has characterized Egypt for decades. The legislative structure erected in Algeria, Morocco, and Tunisia to tackle the COVID-19 crisis, has been more complex and multifaceted, offering many insights. In the first place, all these Maghreb states responded to the health emergency with exceptional legal measures that in general attempted not to be too invasive, and above all, without clashing excessively with their complex legislative frameworks. The result, in some cases, has not been up to Western standards. However, the reality of North Africa and its endogenous democratic deficit over the past decades should be taken into consideration. Within this group of states, each government had its own perception of which legislative measure was most appropriate to cope with the pandemic. That made a difference and highlighted unexpected results. It is quite surprising that Algeria did not revert to a state of emergency, when it had had no hesitation in keeping the nation under a state of emergency, *ḥalāt al-ṭawāri*, for 19 years between 1992 and 2011, even when terrorism was no longer a direct threat.

On the other hand, Tunisia, which in theory has the most advanced political system in transition to democracy after the Arab Spring, became consciously trapped in a never-ending state of emergency, based on an old law passed under Bourguiba’s regime. Another unusual circumstance is that the Tunisian government dangerously mixed a state of emergency with a state of exception, while at the same time relying on strengthening the executive and depriving the parliament of incisive controls. Morocco enacted extremely strict and forward-looking anti-COVID-19 measures. Nevertheless, it was the only country in North Africa to declare a specific health emergency, avoiding a return to the infamous state of exception that had so many negative drawbacks during the reign of Hassan II (1961–1999). Moroccan legislators sought not to impair either the balance of power or the rule of law. If we had to rank these countries in terms of the best result in respect of juridical governance, Morocco would be top the rank order.

In general, we could confirm that Algeria, Morocco and Tunisia followed the COVID-19 guidance issued by the United Nations Office of the High Commissioner for Human Rights on 27 April 2020. Restrictions met the UN requirements as they were provided by law, necessary for the protection of public health, proportionate to the interest at stake, and avoided discriminating against individuals. Moreover, as required, ordinary courts maintained their jurisdiction and did not share their task with military judges, as happened in Egypt. According to the UN guidance, state of emergency legislation should have been strictly limited in scope, the least intrusive to achieve the stated public health goals, and include safeguard clauses in order to ensure the return to ordinary laws. In this case, unfortunately, all three countries were inaccurate in detailing the limits imposed by the UN. Many legislative rules could be applied in other situations beyond the virus emergency, and there were no safeguarding clauses at all, apart from any time limit. Another weak point has been the specific reference to human rights and freedom of speech. The UN recommends that human rights principles, including transparency, should have guided the state of emergency, and should not have been used to stifle dissent. As we pointed out, only Morocco quoted human rights enshrined in its constitution, and opposition has been heavily controlled in Algeria.

Is there a correlation between good juridical governance and success in limiting the spread of the pandemic? Did measures respectful of human rights and the rule of law help to flatten the curve of contagion, or did authoritarian regimes, with their hardline measures, perform better? Did we take for granted the accuracy of World Health Organization figures? We could conclude that pandemic virulence hit both virtuous legal governance and authoritarian regimes. It made little difference to spreading respect for constitutional values. The factors that mattered were much more related to demographic concentration, the impossibility of significant social distancing, and the state of health care systems. Morocco tops the list in terms of numbers of COVID-19 cases, followed by Egypt. These two countries have taken recourse to legislative measures in very different ways, but the result in terms of levels of contagion was not very different, suggesting that the disease did not care much about human rights and fundamental freedoms.

## Notes

1. Libya is characterized by the absence of a single functional government, and no central security forces that can implement anti-pandemic policies nationwide. For this reason, Libya has been excluded from the comparative study in this article.

2. Until May, 142,000 Egyptian citizens were held in detention for breaching the curfew. https://www.shorouknews.com/news/view.aspx?cdate=30052020&id=2669bfef-b837-47c9-8bc5-df23a0934e6d (in Arabic)

3. This amendment has increased the presence of the military in the Egyptian justice. A 1966 law allowed the president to refer civilians to military courts for felonies that pertain to state security during the State of Emergency (Kassem, 2004).

4. Arabic is the only official language in the Algerian Republic. The official French version, *Journal Officiel de la République Démocratique et Populaire* (JORADP), is cited in the text and listed in the references for the reader’s ease. Nevertheless, the Arabic text is the only one deemed authentic and considered to have legal force and as a result, the Arabic versions were used as sources for this article. We applied the same approach with the Moroccan, Tunisian, and Mauritanian journals.

5. For an overall examination of MENA and COVID-19 see Project on Middle East Political Science (POMEPS) (2020).

6. It would have been difficult to declare a state of exception (article 107), because it can be declared only in cases where the country, its institutions, independence, and territorial integrity are in imminent danger.

7. For example, executive decrees were issued on various dates: no. 20-159, 13 June 2020; no. 20-168, 29 June 2020; and 20-182, 9 July 2020.

8. Article 3, executive decree no. 20-168, 30 June 2020.

9. The coalition was formed by the Islamist party of Justice and Development, the Socialist Union of Popular Forces, the National Rally of Independents, and many independent politicians.

10. It must be underlined that the first anti-COVID measure had an economic purpose. The government issued a decree that authorized a special fund, *al-ṣundūq al-ẖāṣ* (الصندوق الخاص), to help the national economy and cushion the social impact of the pandemic (BORM, 2020a).

11. By a bizarre coincidence, the same day, and with the same purpose and juridical reasons, the French government issued *Loi n.* 2020*-290 d’urgence pour fare face à l’épidemie de COVID-19*. Law 2020-290 declared a state of health emergency and authorized the government to take by ordinance any measure adapting the French public procurement code rules to deal with the economic, financial, and social consequences of the spread of the pandemic.

12. Article 7 of 2-20-292 establishes that the decree-law must be ratified by the parliament.

13. Singular *wālī.*

14. The parliament voted the draft law no. 23.20 ‘portant ratification du décret-loi n°2.20.292 du 23 mars 2020 édictant des dispositions particulières à l′état d′urgence sanitaire et des mesures de sa déclaration’. The *Maǧlis al-Nuwāb* unanimously approved the draft law, while the *Maǧlis al-Mustāsharīyyan* did so with just one abstention.

15. Lockdown was lifted on 3 July 2020 by governmental decree 2020-411.

16. Specific sanctions were defined about one month after the curfew was imposed by decree-law 2020/09 on 17 April 2020, which imposed a fine of 50 dinars for violations of the curfew and limitations of movement.

17. On 20 November 2015, the Tunisian Assembly of the People’s Representatives adopted the law on the establishment of the Constitutional Court (*loi organique* n°2015-50). Nevertheless, differences between parties delayed the election of the judges. The elections were due to take place on 8 April 2020, but COVID-19 delayed the procedures again. (Assemblée des Réresentant du Peuple, 2020). The provisional *Instance provisoire chargée du contrôle de la constitutionnalité des projets de loi*, created in 2014, has never been fully operational.
